# Solid–Solid Interfaces in Protonic Ceramic Devices: A Critical Review

**DOI:** 10.1021/acsami.0c13092

**Published:** 2020-12-02

**Authors:** Alessandro Chiara, Francesco Giannici, Candida Pipitone, Alessandro Longo, Chiara Aliotta, Marianna Gambino, Antonino Martorana

**Affiliations:** † Dipartimento di Fisica e Chimica, Università di Palermo, viale delle Scienze, I-90128 Palermo, Italy; ‡ Istituto per lo Studio dei Materiali Nanostrutturati (ISMN)-CNR, UOS Palermo, Via Ugo La Malfa, 153, 90146 Palermo, Italy; § European Synchrotron Radiation Facility, 71, avenue des Martyrs, Grenoble, F-38000, France

**Keywords:** protonic ceramic cells, PCFC, H-SOFC, H-SOEC, H-SOC, solid−solid interfaces, advanced characterization, ab initio modeling

## Abstract

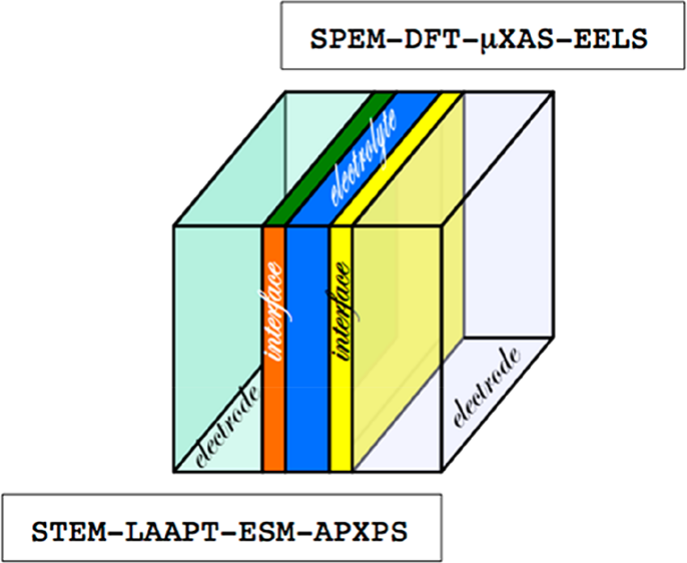

The literature concerning protonic ceramic devices is critically reviewed focusing the reader’s attention on the structure, composition, and phenomena taking place at solid–solid interfaces. These interfaces play a crucial role in the overall device performance, and the relevance of understanding the phenomena taking place at the interfaces for the further improvement of electrochemical protonic ceramic devices is therefore stressed. The grain boundaries and heterostructures in electrolytic membranes, the electrode–electrolyte contacts, and the interfaces within composite anode and cathode materials are all considered, with specific concern to advanced techniques of characterization and to computational modeling by ab initio approaches. An outlook about future developments and improvements highlights the necessity of a deeper insight into the advanced analysis of what happens at the solid–solid interfaces and of in situ/operando investigations that are presently sporadic in the literature on protonic ceramic devices.

## 1 Introduction

It is a fact that, although proton-conducting solid oxide cells (H-SOC) represent in principle the most rational architecture for intermediate-temperature solid oxide fuel cell (SOFC)^1^ and solid oxide electrolysis cell (SOEC)^2^ applications, the oxide ion-conductor devices (O-SOC) are closer to technological maturity. However, the latest achievements of power density for H-SOFCs are becoming comparable to O-SOFCs,^3−6^ and also in the field of high-intermediate temperature electrolyzers, where O-SOEC are reaching the level of technological exploitation,^7,8^ the interest in the development of H-SOECs^9,10^ and of reversible cells based on proton-conducting electrolytes^11^ is steadily increasing.

Boosted by this positive trend, researchers’ creativity aims at further improvements to overcome the issues of protonic ceramics toward the development of effective energy materials. The issues of grain boundary resistivity, electrolyte stability, and chemical reactivity of the electrode–electrolyte couples have been long recognized as crucial^12,13^ and are still pivotal for the development of proton-conducting electrolyzers^9,10^ and proton-conducting fuel cells,^14,15^ together with the quest for electrode materials specifically tailored for proton conducting electrolytes.^16−20^

Regarding what concerns proton-conducting ceramic electrolytes, most of the research activity is focused on the grain-boundary (GB) resistivity, which severely hampers the overall conductivity of electrolytes based on BaCeO_3_ (BCO) and BaZrO_3_ (BZO), but constitutes a drawback also in less exploited materials.^21^ Acceptor-doped BZO, due to its better chemical stability, is usually preferred to BCO in device fabrication. It shows however a wide gap of two orders of magnitude between bulk and grain boundary conductivity at operative temperatures.^22^ The high grain boundary resistance is then tackled by various strategies, which include the use of sintering aids to decrease the densification temperature, pursuing new device fabrication pathways, or exploiting new formulations, possibly involving heterostructures. In the electrode compartment, the most common fuel electrodes are Ni-cermets constituted of NiO mixed with the same oxide used for the electrolytic membrane. Alternative strategies for all-ceramic anodes or composites produced by in situ exsolution of metal particles from the oxide matrix are also pursued. However, the research activity on electrodes is mainly addressed toward the development of suitable air electrode materials, as this is the bottleneck of the device performance. The formulation of specially designed air electrodes points to the development of triple-conducting materials, able to act as catalysts and to conduct protons, electrons, and oxide ions. This goal can be achieved by single-phase compounds, by composites assembled with electrolyte oxides and traditional cathodic SOFC compounds, or by composites with specific formulations, sometimes produced by a one-pot synthesis.

The research activity in these briefly outlined fields was exhaustively reported in recent reviews.^23,14,9,10,15,24−26^ Rather than giving a further general account on H-SOC studies, this paper aims at drawing the reader’s attention to the advisability of investigations, carried out by advanced experimental and modellistic approaches, of what happens at the interfaces between the different components of a proton-conducting solid oxide device. Actually, the effective design of new materials and of device architectures cannot disregard the segregation of dopants and the interdiffusion of cations, eventually resulting in the growth of interface phases, the atomic structure and composition at electrolyte grain boundaries, and the mechanisms governing the transport of protons at interfaces. Then, the review is focused on the solid state contacts, whereas the interfaces of electrodes with the reactive gases are not considered, except for some reference to the overall performance of relevant devices.

The properties of solid–solid interfaces constitute a crucial topic also in other fields of research, and in particular for all-solid state batteries. Cathode–electrolyte, anode–electrolyte, and interparticle interfaces in this field are investigated by computational modeling and experimental techniques, making reference also to in situ/operando approaches; it is worth noticing that the pivotal issues highlighted in this review, such as advanced characterization of interfaces, real time investigations, and modeling of interface processes, are shared with the recent literature on batteries and recently reviewed;^27,28^ these papers could provide stimulating cues for the research activity on protonic ceramic devices.

The sections of this review account for the most representative case studies aiming at obtaining a deeper insight into the structure and phenomena occurring at the interfaces of electrolytes, divided into electrolytes (section 2: Electrolyte Grain Boundaries and section 3: Heterointerfaces in Electrolytes), anodes (section 4: Anode Interfaces), and cathodes (section 5: Cathode Interfaces). The lack of investigations of protonic ceramic materials under operation is highlighted in the short section 6: In Situ/Operando Studies and, finally, in section 7: Concluding Remarks, we draw some overall considerations and focus on specific questions for the future development of protonic ceramic solid oxide electrochemical devices.

## 2 Electrolyte Grain Boundaries

Grain boundaries (GB) constitute a hindrance for proton transport in polycrystalline proton-conducting oxides. The blocking character of grain boundaries is widely ascribed to an excess of positive charge in the grain boundary core, which induces electron accumulation and depletion of protons, oxygen vacancies, and electron holes in the adjacent space charge zones. This important drawback of proton conductors has been investigated for several years starting from the fundamental papers on GB conductivity by Maier and his group.^29^ A comprehensive review on GB ionic conduction in oxide systems was recently given by Gregori et al.^23^

Among the huge literature studies on GB proton conductivity of oxide materials, we cite some papers^30−33^ concerned with the dependence of the grain boundary width on the dopant concentration and on the uneven distribution of acceptor dopants between GB core and space charge regions, as indicative examples of the relevance of nanometer resolution for the investigation of GBs by direct experimental measurements.

Studies on H-SOFCs grain boundaries were carried out by (scanning) transmission electron microscopy ((S)TEM)^34−36^ and by related elemental analyses, such as energy dispersive X-ray fluorescence (EDX) and electron energy loss spectroscopy (EELS), the latter allowing also some information on the oxidation state of elements; a recent advance in atom probe tomography (APT) gave the opportunity of GB imaging at nanometric resolution.^37,38^ These techniques find a powerful complementary use of structural (X-ray diffraction (XRD), X-ray absorption spectroscopy (XAS)) or analytical probes (low energy ion scattering (LEIS), secondary ions mass spectroscopy (SIMS)) and, of course, of electrochemical impedance spectroscopy (EIS) for the functional characterization. In parallel with experimental approaches, *ab initio* computational modeling can give useful indications about the structure and composition of grain boundaries, and energy barriers for proton transport.

Despite well-known drawbacks, entailing low proton conductivity^39^ and interaction with the electrodes,^40,41^ and perhaps precluding the technological development of electrochemical devices based on LaNbO_4_ (LNO),^42^ alkaline earth-doped lanthanum niobate oxides were the subject of papers involving advanced computational and experimental approaches^43,44,21^ that can be taken as reference for GB investigations.

**Figure 1:**
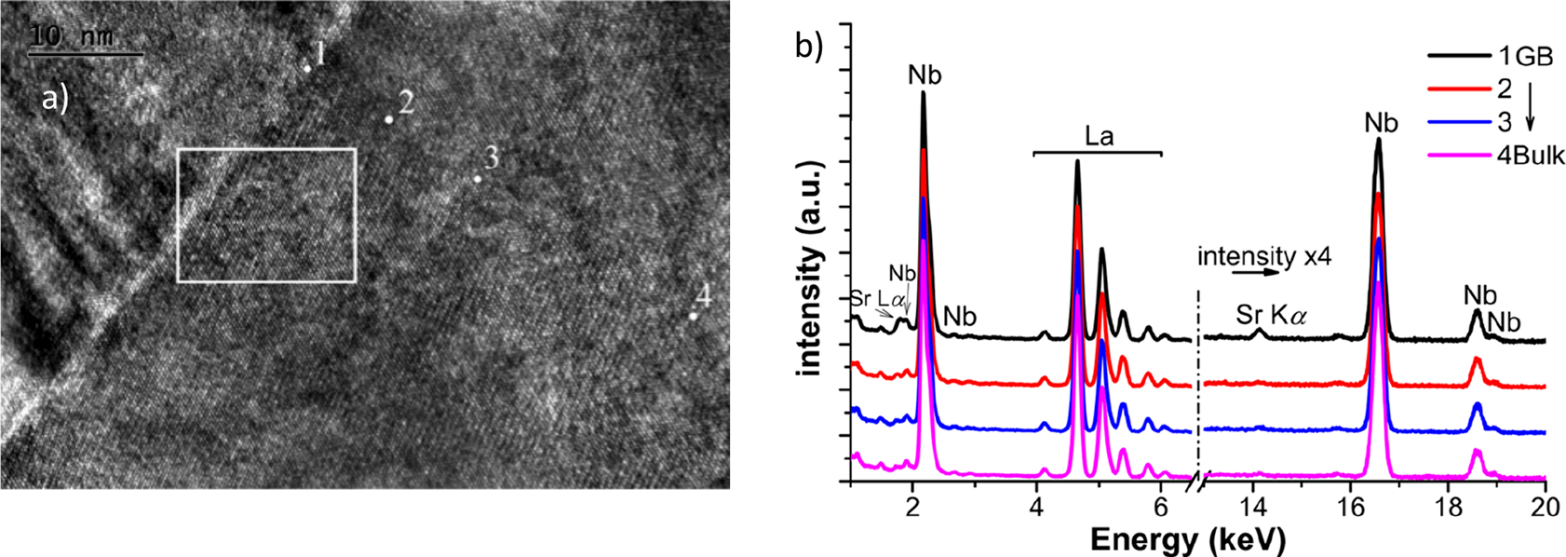
(a) HRTEM micrograph of a La_0.99_Sr_0.01_NbO_4-d_ GB. (b) EDX elemental analyses relative to the spots labeled in (a). Adapted from ref (21). Copyright 2017 American Chemical Society.

In particular, the GB structure of the proton conductor La_0.99_Sr_0.01_NbO_4-d_ (LSNO) was recently investigated by Li et al.^21^ using high resolution transmission electron microscopy (HRTEM) and LEIS. The micrograph of the grain boundary region (Figure 1a) evidenced that the GB was well crystallized, with no amorphous or secondary phase present. The elemental analysis (Figure 1b) was carried out at the spots marked in Figure 1a, giving evidence of an Sr enrichment across the grain boundary and hinting at the increasing concentration of negative Sr_Nb_^′^ defects (in Kröger–Vink notation) going toward the grain boundary core. The positive GB core, only partially screened by the effective negative charge of the dopant, determines the charge carrier depletion in the space charge layer. The LEIS spectra were taken on the surface of the as-sintered sample, on the as-polished surface and after different annealing treatments. The as-sintered sample shows Sr surface segregation that is removed by polishing, exposing a bulk-composition fresh surface, while the successive annealings at different temperatures restore the Sr surface enrichment. In agreement with the mechanism of surface enrichment put forth by Druce et al.,^45^ it is proposed that the Sr diffusion toward the surface proceeds mainly via grain boundary pathways, due to the larger dopant concentration in the GB region. The formation of a Sr-rich surface phase and the reduced concentration of surface oxygen vacancies could have the effect of blocking water intake and impeding proton diffusivity.^46^

**Figure 2:**
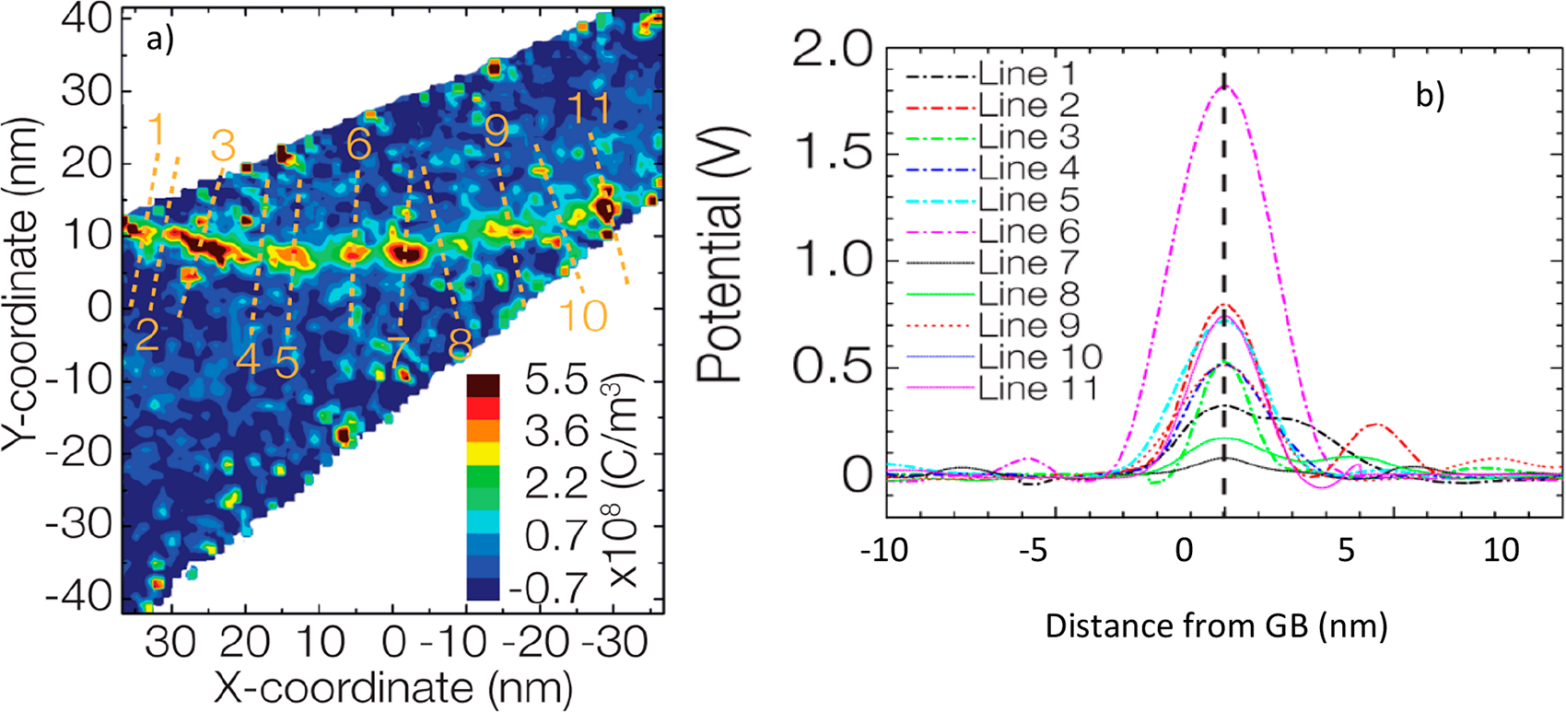
(a) Contour map of the charge distribution in the grain boundary region of the BZY electrolyte. The red-green zone corresponds to the GB region. Red color denotes positive and blue negative charge, according to the reported scale bar. (b) Electrostatic potential across the lines of Figure 2a. Adapted from ref (37). Copyright 2016 American Chemical Society.

In recent studies, laser-assisted atom-probe-tomography (LAAPT) was used to analyze the grain boundary chemical composition of a BZY10 (BaZr_0.9_Y_0.1_O_3-δ_) electrolyte.^37^ In the LAAPT technique, ions from the sample are evaporated by a laser pulse. The time-of-flight of the vaporized species against a microchannel plate is used to reconstruct the three-dimensional composition with sub-nanometric resolution.^47^ In the cited paper,^37^ LAAPT was used to analyze the GB composition of a BZY10 electrolyte and allowed the researchers to demonstrate the segregation of oxygen vacancies at the GB core and of yttrium at GB core and in the space charge region. The charge density distribution was then derived by solving the Poisson–Boltzmann equation for the grain boundary potential. Figure 2 shows the charge distribution in the grain boundary region, giving direct evidence of a positive charge distribution at GB. From the calculated charge distribution, the variation of the electrostatic potential along the lines crossing the GB and drawn in Figure 2a was derived (Figure 2b).

Following this pioneering paper, the atom probe tomography (APT) was recently applied by Burton et al.^38^ to the investigation of grain boundaries in a BaCe_0.8_Y_0.2_O_3−δ_-Ce_0.8_Y_0.2_O_2−δ_ composite membrane (BCY-YDC), where the BCY proton conductor and the YDC oxygen-ion and, in reducing environment, electronic conductor are assembled to fabricate a material for hydrogen separation.^48^ Burton et al. analyzed the three different YDC/YDC, BCY/BCY, and BCY/YDC phase boundaries within the YDC-BCY membrane and measured various GBs samples for each phase boundary type, reconstructing the respective 3D structure and GB composition. The observed differences between the samples allowed the researchers to conclude that the strategies for GB conductivity improvement should be accompanied by suitable correlation between the macroscale electrochemical behavior with the nanoscale structural/compositional analysis in order to tailor materials with improved performances. Among the strategies adopted to mitigate the GB core positivity, we cite the annealing treatments to increase the concentration of negative acceptor dopant defects in the space charge region^49,34^ and GB decoration by acceptor dopants;^50^ in fact, these attempts and the other similar cases with oxide-ion conductors (e.g.,^51,52^) should require suitable investigations in order to monitor the structure and composition of the GBs engineered in this way and their stability in operative conditions.

For an atomic-level understanding of the segregation of charged defects at the GB core and of the consequent mobile charge carrier depletion in the adjacent space charge region, the experimental approaches described above were complemented by computational modellization of grain boundaries. Starting from the consideration that the GB proton resistivity in barium zirconate is an intrinsic feature of the oxide, Helgee et al.^53^ calculated by DFT the segregation energies of oxygen vacancies and of proton defects in three different symmetric BZO GBs, finding segregation energies ranging from −0.5 to −1.5 eV, depending on the core GB plane. These values were used as input to the space charge layer (SCL) model, obtaining the charge profiles and the electrostatic potential as a function of the distance from the core GB plane.

**Figure 3:**
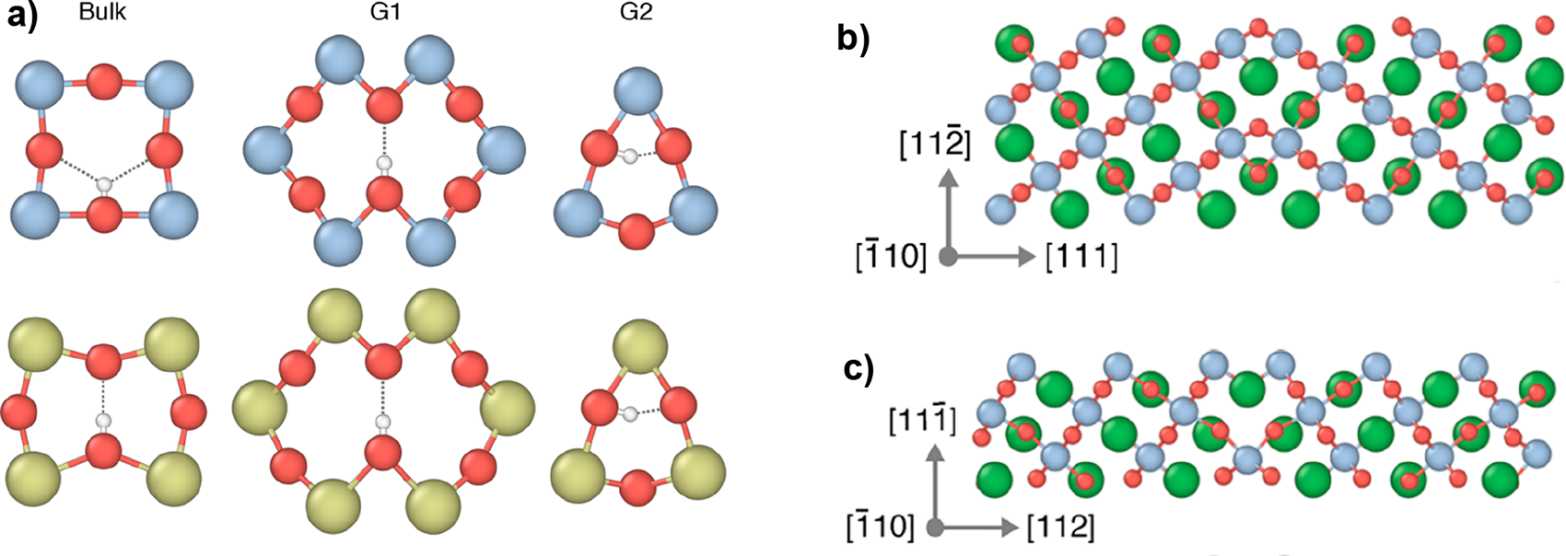
(a) Local environment of protons in bulk and GBs. Upper drawings are relative to BZO, lower drawings to BCO (Zr blue, O red, Ce green): “Bulk” labels bulk proton configurations of BZO and BCO; “G1” labels the GB proton configurations relative to the symmetric boundary drawn in (b); “G2” labels the GB proton configurations relative to the symmetric boundary drawn in (c). (b) and (c) refer to the cubic cell of BZO; for the BCO tetragonal unit cell the G1 and G2 configurations spread into several similar configurations. Adapted from ref (22). Copyright 2017 American Chemical Society.

Similar calculations were reported in other papers,^54,55^ and in particular an interesting interpretation of the different grain boundary resistivity in BCO and in BZO was given.^22^ In this paper, it is reported that protons are more stable in bulk BCO, with a formation energy of about 0.4 eV lower than in BZO; this difference is mainly ascribed to the higher flexibility of the BCO network, allowing for interoctahedral nearly linear and shorter hydrogen bonds, whereas these bonds are bent, longer, and intraoctahedral in BZO (Figure 3a, “Bulk” drawings). On the other hand, the insertion of protons in the distorted GB region does not require major structural rearrangements (Figure 3a, “G1” and “G2” drawings), and therefore the elastic properties of the two oxides become less important. Then, the strained GB structure leads to a similar proton local environment for BCO and BZO. As a consequence, the proton segregation energy, calculated as the difference between the formation energies in the GB and in the bulk:
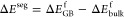
1becomes more negative for BZO, due to the definitely lower proton defect formation energy in bulk BCO.

A modellistic approach to GB analysis was pursued also by Polfus et al.,^56^ who calculated the defects segregation energies at the (021)[100] symmetric tilt boundary of Y-doped barium cerate. The considered defects were V_O_^••^, OH_O_^•^, Y_Ce_^′^, Ce_Ce_^′^, and (V_O_^••^ Ce_Ce_^′^)^•^, and in particular electron defects were treated as localized Ce^3+^ small polarons strongly bound to oxygen vacancies. According to the authors’ speculation, these defects may acquire a relevant role of n-type grain boundary conduction under a reducing environment in Ce-containing electrolyte formulations.

In the effort to overcome the grain boundary issues of BaZrO_3_-related materials, sintering aids were exploited since the early 2000s^57^ until today.^58−60^ Very recently, Luo et al.^36^ reported electrical measurements showing a definite increase of conductivity with respect to the membrane formed with BZY alone; these improvements were correlated to the lower sintering temperature allowed by the sintering aids, that limits Ba loss and promotes grain growth and densification. On the other hand, the very recent mini review by Li et al.,^25^ with the eloquent title: “Sintering aids for proton-conducting oxides: A double-edged sword?” raises the question about the real suitability of sintering aids to overcome the poor sinterability of barium zirconate-based membranes. Actually, the debate is presently open, and the evidence for and against sintering aids is contradictory, depending on the overall appraisal of the device performance or on the assessment of the electrolyte properties. The opposition party, in particular, claims that the sintering aid decreases water uptake and proton conductivity of BZY electrolytes, while increasing hole conductivity.^61^ This debate concerns also the “solid state reactive sintering” (SSRS), consisting of adding a small amount of sintering aid, usually NiO, in the mixture of the precursors of the electrolyte membrane and carrying out directly the sintering procedure.^62^ The question was recently addressed by Huang et al.,^63^ who compared the behavior of three different BZY membranes, prepared by (i) SSRS, (ii) spark plasma sintering of BaCO_3_ + YSZ as a NiO-free reference, (iii) traditional solid state synthesis starting from the single oxides BaO, ZrO_2_, Y_2_O_3_, and NiO. The hydrated samples were subjected to thermogravimetric analysis to determine the amount of water uptake, finding that the addition of NiO decreased the capacity of water uptake and then the concentration of charge carriers. Moreover, EIS data evidenced an increased grain boundary resistivity. The most probable Ni locations were hypothesized in grain boundary phases such as BaY_2_NiO_5_ and BaNiO_2_, while a lower probability was attributed to interstitial defect clusters V_Ba_′′Ni_i_^••^ within the barium zirconate matrix; in all these cases, the incorporation of Ni involved annihilation of oxygen vacancies and then a decreased concentration of OH_O_^•^ defects. Some indication about the fate of Ni in BZY came from the XAS experiments performed by Han et al.,^64^ demonstrating the formation of the cluster V_Ba_′′Ni_i_^••^ and, by XANES spectroscopy, that the average valence of Ni was lower than 3+. This last result is coherent with the increased lattice constant of BZY as a function of the oxidation state of Ni determined by XRD, implicitly confirming that Ni is also inserted in the BZY matrix; the authors argue that the excess Ba is likely driven to the GB, where an amorphous Ba-containing phase boosts the sintering of BZY grains.

The detrimental effect of sintering aids can even take place due to interdiffusion of cations coming from anode and cathode materials used in the assembly of H-SOCs, as demonstrated by Han et al.^65^ These authors introduced Mn, Co, and Fe into the BZY matrix by a solid state reaction and observed the interdiffusion by XANES spectroscopy; then, electrochemical analysis proved the decrease of proton conductivity and proton transport number with respect to pure BZY. A similar conclusion was drawn by Shimada et al.,^66^ who demonstrated by field emission-electron probe microanalysis (FE-EPMA) the diffusion of Ni into the BCZYYb (BaZr_0.1_Ce_0.7_Y_0.1_Yb_0.1_O_3−δ_) matrix originated in an anode-supported cell by cosintering of the BCZYYb/Ni-BCZYYb electrolyte/anode couple. Also in this case electrochemical analysis evidenced the degradation of proton conductivity. Notably, a not so different route, consisting of a Ni-BaCe_0.55_Zr_0.3_Y_0.15_O_3-δ_ anode-assisted densification of a 5 μm-thick BaCe_0.55_Zr_0.3_Y_0.15_O_3-δ_ electrolyte, gave an excellent power density peak of 1300 mW/cm^–2^ at 600 °C.^67^ Good results, as concerns the introduction of sintering aids, were achieved also by Yang et al.,^68^ who prepared by a Pechini method an electrolyte with composition BaCe_0.68_Zr_0.1_Y_0.1_Yb_0.1_Cu_0.02_O_3−δ_ (BCZYYC2) and assembled a reversible Ni–BCZYYC2/BCZYYC2/LSN–BCZYYC2 SOC device exhibiting remarkable electrochemical performance and stability in fuel cell and electrolyzer operation modes.

Among the strategies to overcome grain boundary issues, the idea of eliminating grain boundaries by a columnar architecture of the BZY layer deserves a citation;^69,70^ the latter study reached a peak power output of 740 mW/cm^–2^ at 600 °C with an anode-supported BZY layer 2.5 μm thick grown by pulsed laser deposition (PLD).

**Figure 4:**
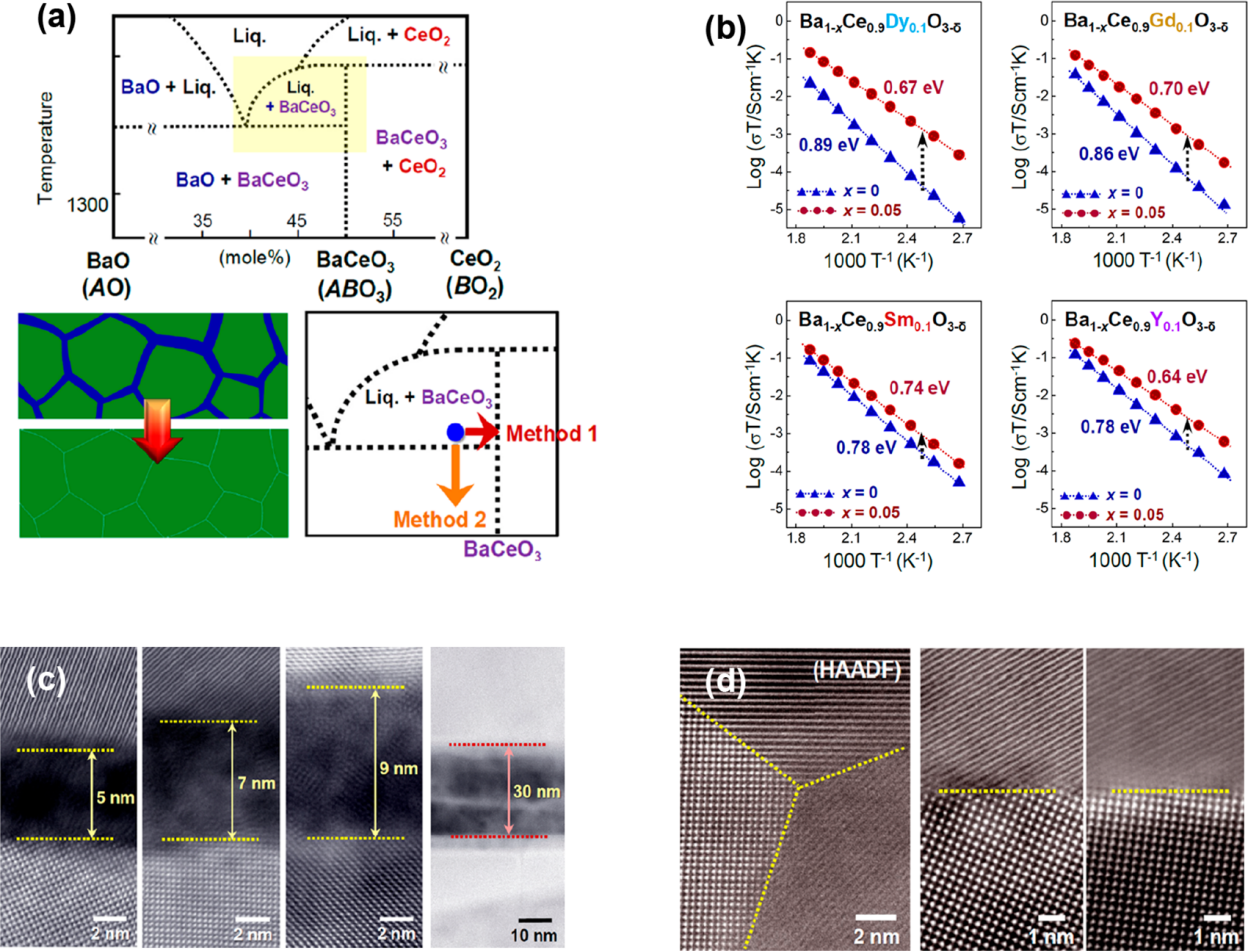
(a) BaO-CeO_2_ phase diagram; the yellow zone is relative to the coexistence of BaCeO_3_ + a Ba-containing amorphous phase; (b) conductivities and energy barriers in correspondence of the stoichiometric and substoichiometric Ba content; (c) TEM micrographs of the amorphous intergrain phase; (d) grain boundaries in correspondence of substoichiometric Ba content. Adapted from ref (71). Copyright 2018 American Chemical Society.

Finally, it is worth noticing that barium cerate-based materials could partially overcome their issues of chemical reactivity by using straightforward recipes. Kim et al.^71^ outlined two approaches, based on the BaO-CeO_2_ phase diagram (Figure 4a) and on a careful analysis of the composition and structure of BaCeO_3_ grain boundaries (Figure 4c). In Figure 4a, the yellow zone corresponds to the simultaneous presence of BaCeO_3_ and an amorphous Ba-containing phase; the latter, with varying thicknesses, is recognizable in the GB micrographs of Figure 4c, relative to a barium cerate sample sintered at 1400 °C. It was demonstrated that this Ba-rich amorphous phase is originated by a 3–5 atom % of Ce in the A perovskite site, leading to an excess Ba amount and to its rearrangement in the GB amorphous phase, if the ideal 1:1 stoichiometric composition of BaCeO_3_ is fulfilled in the oxide synthesis. To avoid the formation of the intergranular amorphous phase, which is responsible for the infiltration of H_2_O and CO_2_ producing the failure of the compound, two methods were proposed. The first method is to prepare the oxide with a slight Ba deficiency, and the second is to perform sintering at 1400 °C followed by a lower temperature annealing at 1150 °C. Both recipes allow getting out of the yellow zone in Figure 4a and lead to the drastic reduction, or even elimination, of the amorphous region. Correspondingly, an improved GB conducibility is attained, and the energy barrier for proton diffusion is lowered (Figure 4b).

In summary, the direct observation of GB structure in protonic electrolytes is challenging and not frequently pursued; the ab initio computational approaches are rarely pursued too, but they can provide hints about the dependence of the grain boundary resistivity on the mutual orientation and atomic structure of contacting grains and, perhaps, an outlook on the strategies for overcoming carrier depletion in the space charge layer. Structural techniques such as XRD and XAS, in synergy with high resolution microscopy, can provide the necessary information for an assessment of materials synthesis and device engineering that are often left to the mere appraisal of the overall device performance.

## 3 Heterointerfaces in Electrolytes

The close contact between different materials can give rise to novel properties that can be tailored by suitable interface engineering. So, epitaxial growth of thin layers on an underlying crystal facet and multilayered or vertically aligned architectures can enhance, in the field of electrochemical devices, ionic conductivity and electrocatalyst activity. These peculiar properties can be achieved exploiting various effects, ranging from modified electronic structure, strain induced by the misfit between different lattices, or asymmetric space charge taking place due to the different composition of the facing materials. A comprehensive review on heterostructures in electrochemical solid oxide devices was recently authored by Zhao et al.^26^

**Figure 5:**
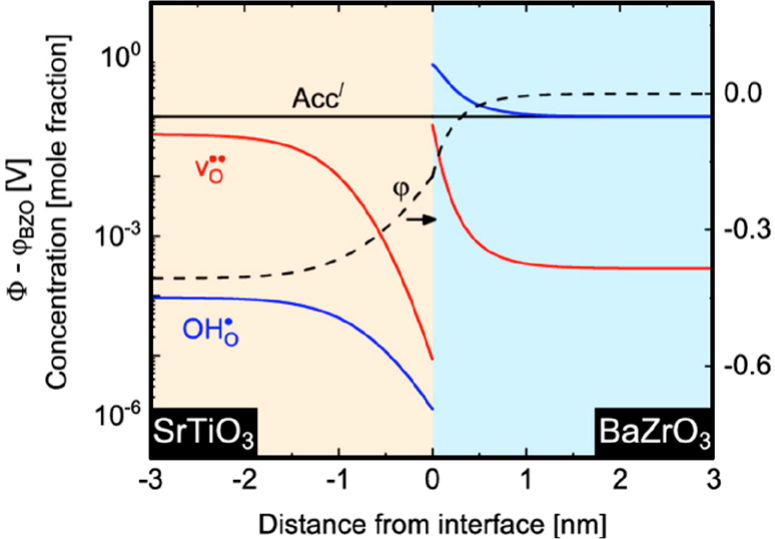
Charge carrier concentration (red: V_O_^••^, blue: OH_O_^•^) and potential as a function of the distance from the BZO/STO interface at 600 K; the dopant acceptor concentration is kept constant, according to the Mott–Schottky model. Adapted from ref (73). Copyright 2019 American Chemical Society.

The heterointerfaces in proton conductors have been investigated by both advanced experimental techniques and by computational modeling. Polfus et al.^72^ studied the heterostructure BaZrO_3_(001)/MgO(001), which displays a small mismatch of 0.25% between the periodicities of the two oxides, focusing the DFT modeling on the space charge depletion originated by the positively charged interface. A similar approach was applied by Saeed et al.^73^ to the study of the heterointerface BaZrO_3_/SrTiO_3_ (BZO/STO). By ab initio DFT calculations, these authors demonstrated that the negative segregation energies of V_O_^••^ and OH_O_^•^ in BaZrO_3_ with respect to SrTiO_3_ determines the formation of a positive potential on the BZO side (Figure 5). Then, the related depletion layer takes place on the side of STO, which can act as an effective acceptor dopant for BZO. The increased charge carrier concentration on the BZO side is eventually at the origin of its enhanced protonic conductivity.

The heterointerface BaZrO_0.8_Y_0.2_O_3−δ_/(110)NdGaO_3_ (BZY/(110)NGO) was investigated in a series of papers by experimental and computational approaches. The strong mismatch of about 10% between the two surfaces was correlated with the proton conductivity measured by in-plane EIS. The interface structure and composition was investigated by STEM/EELS,^74^ showing the high dislocation density relieving the strain originated by the mismatch of BZY with (110)NGO; the EELS maps evidenced Ba depletion and Zr enrichment on the BZY side close to interface. The high proton conductivity measured by in-plane EIS (about two orders of magnitude larger than typical BZY bulk conductivity) was correlated to the distorted interface structure using electrochemical strain microscopy ( ESM). The latter technique uses the tip of AFM to induce a local response from a material surface, that is measured as a local strain. When a proton conducting film is probed, the positively biased tip injects into the sample protons coming from the dissociation of the water meniscus at the tip; at the negative bias the reverse reaction takes place, and in both situations the instrument measures the induced local strain.

**Figure 6:**
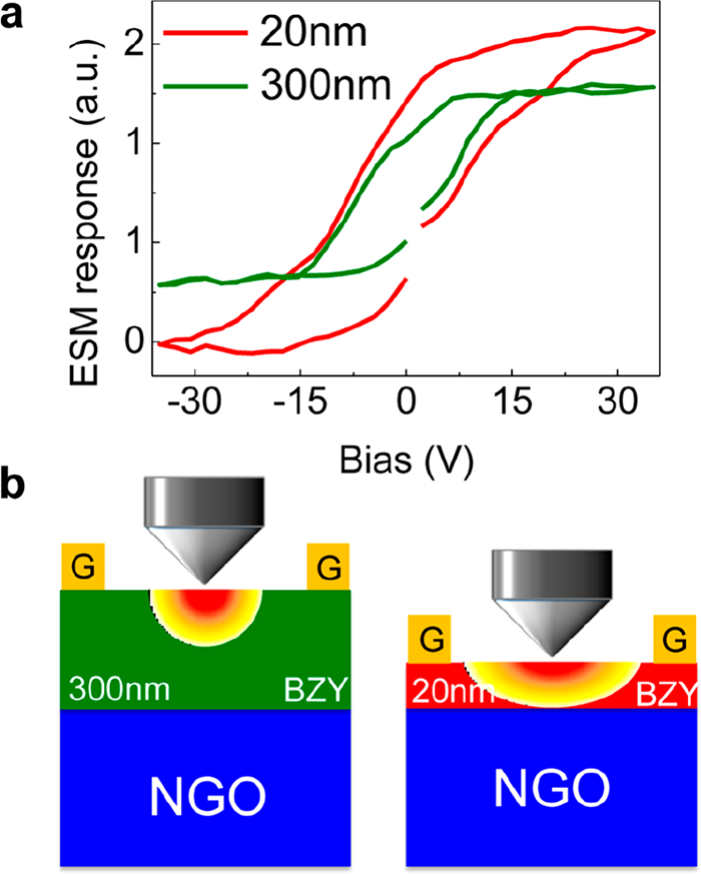
(a) ESM hysteresis loops relative to the response of the two probed BZY/(110)NGO heterointerfaces with a BZY film thickness of 300 and 20 nm, respectively. (b) Scheme of the experiment: the AFM tip scans the surface of the two films with a penetration depth of about 20 nm, so the thinner film is probed close to the interface. Reproduced from ref (74). Copyright 2015 American Chemical Society.

Figure 6 shows the scheme of the experiment, applied to a 300 nm and, respectively, to a 20 nm thick films. The hysteresis loops are different: while the hysteresis of the thick film saturates at ±15 V, the thin film does not saturate. The different behavior is related to the local strain that induces higher proton conductivity in the interfacial region and then facilitates in the thinner film the relaxation of the local perturbation produced by the tip.

In a subsequent paper by the same group,^75^ a 30 nm thickness BZY film was deposited onto the (110)NGO substrate by PLD and analyzed by high energy photoelectron spectroscopy (HAXPES) and STEM-EELS. The latter technique gave evidence of the formation of a columnar region, about 2 × 10 nm wide and extending throughout the BZY film from the substrate until the surface, where a good deal of Ba atoms are substituted by Y^3+^ and Zr^4+^. The authors’ interpretation was that, due to the smaller size of yttrium and zirconium, this substitution was alternative to the formation of dislocations to relieve the compressive strain at the BZY/NGO interface. DFT modeling demonstrated that proton hopping is barrierless in the regions where Y^3+^ substitutes Ba^2+^ and is present also in the octahedra surrounding the substituted A site. So, it was speculated that these columnar channels constitute privileged pathways for proton conduction.

A completely different heterostructure was conceived by Jeong and co-workers,^76^ who deposited by reactive cosputtering a thin (1–3.5 μm) BZCY film on an anode Pd foil. The comparison with a conventional anode-supported cell showed that the metal-supported cell had a very low distribution of oxygen vacancies throughout its width, due to the blocking effect of oxide ions at the Pd anode. Then, the charge neutrality was ensured by an enhancement of protonic defects, pumped into the electrolyte by the hydrogen permeable Pd membrane.

The reported studies are indicative of the different ways of enhancing proton conductivity by different heterointerface architectures: the computational simulations of Polfus and Saeed^72,73^ and the metal-supported cell of Jeong^76^ point to different mechanisms of increasing the protonic defect concentration, while in the papers of Aruta and co-workers^74,75^ the interface interaction produces preferential low resistivity proton pathways. To the best of our knowledge, the papers cited in this section and a few more^77−79^ are the only ones dealing with heterointerfaces in proton conductors, while the implementation of these ideas in operating devices seems presently far from being accomplishedt. On the O-SOC side, the research is definitely more active, producing also specially tailored device architectures.^80−82^

## 4 Anode Interfaces

In most protonic ceramic devices (SOFC mode), the anode is composed by Ni-electrolyte cermets. This choice favors the anode-electrolyte compatibility^40^ but, in particular in cosintered anode-supported devices, determines the diffusion of Ni species into the electrolyte layer. This diffusion, even when unwanted, acts as a sintering aid, an effect involving the contrasting evaluations discussed in section 2.^65−67^

Actually, the anode performance depends also on the fabrication route; for instance, Bae et al.^83^ supported using PLD a thin BCZY layer on a composite anode consisting of a Ni-BCZY cermet with a Ni concentration that decreases on going toward the electrolyte. The so-prepared anode enhanced the mechanical stability of an electrolyte layer 2 μm thick, and allowed a good peak power of 635 mW/cm^–2^ at 600 °C to be obtained.^83^ With the aim of avoiding Ni-poisoning of the electrolyte, the same group^84^ fabricated an anode-supported device consisting of a Ni-YSZ composite, performing the tasks of electronic conductor and of a mechanically and chemically stable support. On this support, a thin layer of Ni-BCZY ensuring the H^+^ transfer to the electrolyte and, in sequence, the electrolyte and a BSCF conventional cathode, were deposited by PLD. The electrolyte deposition was carried out at a temperature of 750 °C, so limiting the diffusion of Ni toward the electrolyte. Following the strategy of blocking the diffusion of Ni into the electrolyte layer, Han et al.^85^ adopted a device fabrication consisting of the infiltration of the Ni particles into the BZY porous backbone of the anode, which was previously cosintered with the electrolyte at high temperature, while Anggia et al.^86^ cut it short proposing an all-ceramic Ni-free (La_0.8_Sr_0.2_)(Cr_0.5_Mn_0.5_)O_3−δ_–Ba(Zr_0.75_Y_0.15_)O_3−δ_ composite.

**Figure 7:**
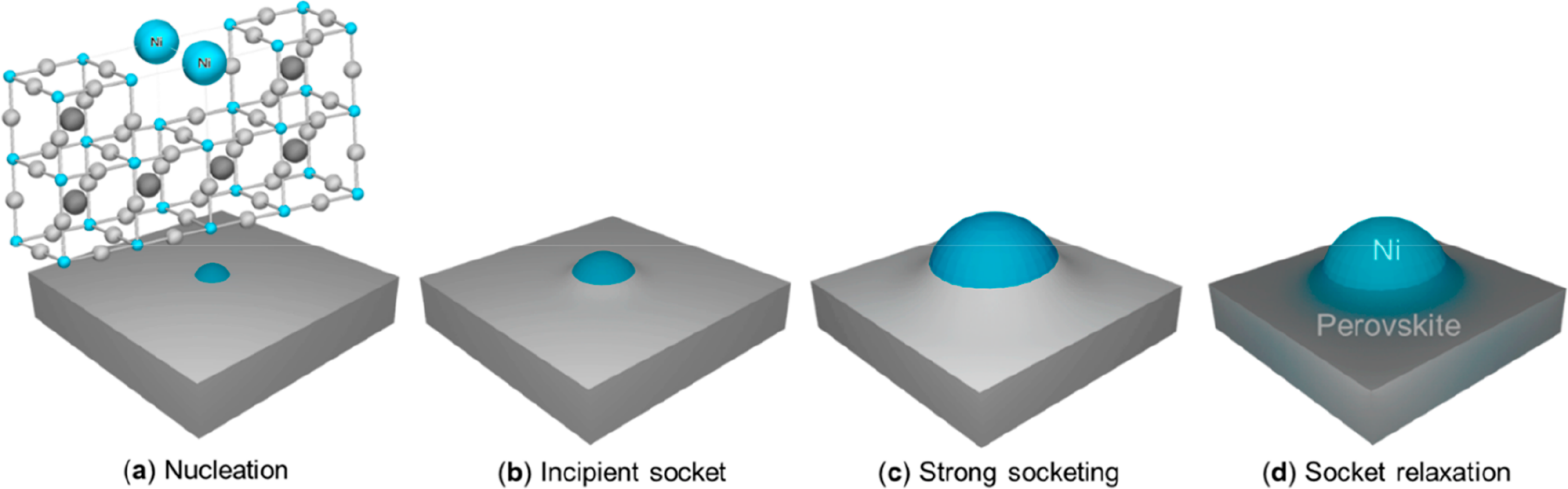
(a) Nucleation of the exsolved particle, seeded by Ni dopants segregated from the perovskite B-site; (b) the Ni particle grows pushing and lifting the perovskite lattice; (c) a volcano-shaped nanostructure is formed around the grown particle; (d) the volcano relaxes, but the Ni particle keeps a strong contact with the oxide matrix. Reproduced from ref (90). Copyright 2019 American Chemical Society.

An alternative way of tailoring anodic materials is the exsolution of metal particles from a perovskite oxide of general formula ABO_3_. The technique is based on the early studies of Irvine and collaborators, who found that A-site deficiency could trigger the exsolution of B-site species under reductive environment.^87^ The exsolved particles are partially immersed, or socketed, in the oxide surface and act as a supported catalyst, with several interesting properties such as control of size, homogeneous distribution, reversibility of exsolution under oxidation, strong interaction with the support ensuring stability, and, most important, synergistic interaction with the oxide.^88,89^ A scheme of the surfacing of a Ni particle from a perovskite is pictorially described (Figure 7) in a recent paper by Neagu et al.^90^

An example of Ni-BZY anode obtained by exsolution of the Ni particles is given in the paper by Duan et al.,^91^ who used the SSRS procedure to assemble a cell with very good properties of durability and tolerance to poisoning. The authors propose a scheme to explain how the synergistic interaction between the BZY support and the Ni particles improves the stability of the cell with different carbonaceous fuels, without anode coking or sulfur poisoning. In summary, carbon cleaning and desulfurization take place thanks to the intimate contact between the exsolved Ni particles and the BZY support, allowing a facile reaction between the poisoning species bound to the catalyst and the hydroxyls coming from the dissociative water adsorption on the BZY surface.

The exsolution of metal particles was also exploited for the anode of a proton conducting electrolyzer cell for the dehydrogenation of ethane to ethylene.^92^ A coking free anode material is clearly essential for this process: it was constituted of Ni_*x*_Cu_(1–*x*)_ metal particles exsolved from a Nb_1.33_(Ti_0.8_Mn_0.2_)_0.67_O_4−δ_ (NTMO) oxide support. The strong interaction with a model TiO_2_ support was investigated by a computational DFT approach, showing in particular that the alloy Ni_0.5_Cu_0.5_ has a composition that favors the activation of the C–H bond. The analysis of the reaction products confirmed the beneficial effect of the strong metal–support interaction in terms of catalytic yield, absence of coking, and catalyst stability.

In summary, the anode-electrolyte interfaces, and those between the different components of a composite anode material, are important for the performance and durability of the anode compartment. In most cases, the SOFC anode is a composite Ni-electrolyte, so the main issue to tackle is the diffusion of Ni, either toward the separating membrane, or within the anodic material. This goal can be achieved by suitable electrode fabrication. On the other hand, exsolved metal particles constitute a very nice example of chemical/morphological stability and of the synergistic effect between the various components of a composite anode. These materials are recognized as examples of strong metal–support interaction (SMSI) in the field of electrochemical devices, with promising prospects for further improvements.^93^ In this respect, computational modeling of the composite and operando studies could yield useful cues for future development.

## 5 Cathode Interfaces

The effective contact at the cathode/electrolyte interface is crucial for the operation of electrochemical devices. For protonic ceramic cells, the quest for specific cathodes overlaps with the issue of their interaction with electrolytes, so that synthesis of new materials and device engineering are both challenging and closely correlated.

The analysis of what happens at the interface between proton-conducting electrolytes and electrodes is necessarily related to the different device fabrication procedures and to the specific electrolyte-electrode couples. The assembly of a proton-conducting oxide with air electrodes already designed for O-SOC devices was initially pursued^94−97^ and continues to this day.^98−100^ A straightforward method to assess the electrode–electrolyte reactivity is by mixing the two compounds, forming a pellet to ensure the closest contact, annealing at a suitable temperature for a given time, and then carrying out an XRD measurement. This procedure is frequently exploited: in some cases, a shift of diffraction peaks is observed, originated by diffusion into the host matrix of cations with a different size compared to the regular species;^101−103^ in other cases, the growth of a new phase may occur,^104,100^ or the stability of the involved oxides is eventually demonstrated.^105^ The general validity of these conclusions should be carefully assessed, since these XRD analyses do not investigate the actual interface between the device components and, moreover, suffer from limited sensitivity to amorphous phases and trace compounds.

A deeper insight into H-SOCs cathode–electrolyte interface was recently carried out by De Vero et al.^106^ in a study investigating the poisoning of LSCF (La_0.6_Sr_0.4_Co_0.2_Fe_0.8_O_3−δ_), the most common O-SOC cathodic material, by atmospheric sulfur dioxide. An LSCF thin film was grown by PLD on GDC (Gd_0.1_Ce_0.9_O_1.95_) and, respectively, on BZY; then, the two samples were annealed for 300 h at 800 °C in the presence of trace amounts of sulfur, and the cross sections of the two cathode-electrolyte interfaces were analyzed by STEM-EDX maps and SIMS depth profiles. These techniques allowed researchers to evidence a LSCF-BZY massive cation interdiffusion, producing also Kirkendall pores within the LSCF layer; on the other hand, a chemically inert interface was proven between GDC and LSCF.

Another comparative study of reactivity between a “traditional” cathodic material (LSM, La_0.8_Sr_0.2_MnO_3–*x*_) with a proton conductor (BCY, BaCe_0.9_Y_0.1_O_3–*x*_) and an oxide ion conductor (SDC, Ce_0.8_Sm_0.2_O_2–*x*_) was carried out by Giannici et al.^107^ The two interfaces LSM-BCY and LSM-SDC were investigated by X-ray microspectroscopy. This synchrotron technique has a submicron lateral resolution, obtained by mirror focalization of the X-ray beam and allows the acquisition of micro X-ray fluorescence (μ-XRF) raster scans of a sample region at different incident energies;^41,108,109^ then, the technique offers also the opportunity to perform micro X-ray absorption near-edge structure (μ-XANES) scans on small regions of the sample, so providing data on the oxidation state and local atomic structure of the X-ray absorbing species.

**Figure 8:**
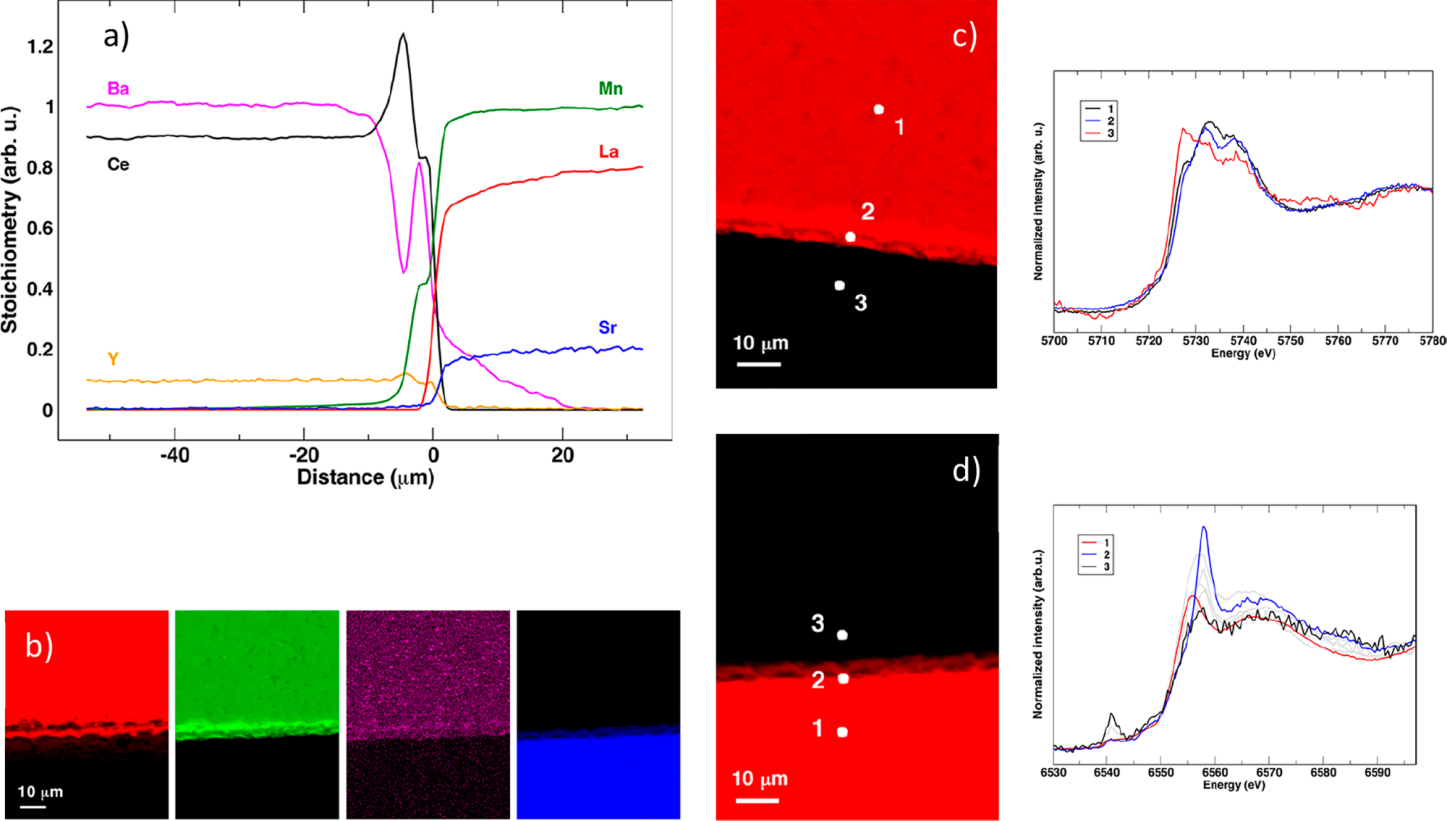
(a) Concentration profiles relative to elements of the BCY-LSM couple. (b) Color maps relative to Ba (red), Ce (green), Y (violet), and Mn (blue). (c) Ce L_3_-edge μ-XANES spectra taken at the points indicated in the map. (d) Mn K-edge μ-XANES spectra taken at the points indicated in the map. Adapted from ref (107). Copyright 2019 American Chemical Society.

X-ray microspectroscopy gave evidence of a high chemical reactivity of the couple LSM-BCY and of longer range cation interdiffusion paths in comparison with LSM-SDC.^107^ The concentration profiles shown in Figure 8a, relative to the LSM-BCY couple, show the formation of interface phases after 72 h annealing at 1150 °C, a process that was already observed, to a lesser extent, after 12 h treatment. Going from left to right in Figure 8a, a depletion of Ba and an increase of Ce gives rise to a Ce and Y-rich layer, followed by a Ba–Mn and, in sequence, by a Ce–Y layer. The μ-XANES spectra us allow to interpret the elemental analysis in terms of local structures and oxidation states of cerium: Ce^4+^ in octahedral coordination in bulk BCY (spot 1 in Figure 8c), Ce^4+^ in a doped cerium oxide environment (likely YDC, spot2 in Figure 8c), a partially reduced oxidation state Ce^4+^/Ce^3+^ in bulk LSM, (spot 3) where it is hosted in the A-site of the LSM perovskite and substitutes La. The fate of Mn is even more interesting: it starts in octahedral coordination and oxidation state 3+ in LSM (spot 1 in Figure 8d); then, in the interface region (spot 2), μ-XANES features are relative to Mn^4+^ in octahedral environment that, taking into account the Ba interface enrichment, is likely related to a BaMnO_3_ local structure; finally, in bulk BCY (spot 3), the μ-XANES data are coherent with Mn^6+^ in a tetrahedral environment, likely a BaMnO_4_ local structure.

The two case studies presented show the great potentiality of advanced characterization techniques applied to H-SOC materials, and also highlight the need for specially designed electrode materials for H-SOCs to optimize performances but also to overcome cation interdiffusion that can even give rise to the growth of interface phases, eventually producing device degradation and/or delamination. In fact, there is a wide agreement that a major hindrance for the development of H-SOCs is represented by the lack of suitable electrode materials, particularly cathodes,^110,20,111^ whose operation is crucial for tailoring effective H-SOC devices.^15^

The specially designed cathodic materials for H-SOCs can be composites,^20,18,19,112−114,6,115,116^ or single-phase triple-conducting oxides (TCO) capable of the simultaneous transport of O^2–^, e^–^, and H^+17^.^117−125,16^ Some of the latter compounds are simple^117,121,122,17^ or double perovskites,^118−120^ other are Ruddlesden–Popper layered perovskites.^123,125,126^ Zohourian et al.^16^ investigated by thermogravimetry the proton uptake of several (Ba,Sr,La)(Fe,Co,Zn,Y)O_3-δ_ perovskites as potential cathode materials, giving general rules for the design of cathodic oxides for H-SOFCs.

Among single-phase TCO compounds, Xia et al.^17^ achieved, using BaFe_0.-8–*x*_Sn_0.2_Bi_X_O_3−δ_, the highest power density ever obtained (650 mW cm^–2^ at 550 °C) with a cobalt-free cathodic material. This perovskite oxide was designed as a modification of the SOFC MIEC oxide BaFe_0.95_Sn_0.05_O_3−δ_, adding bismuth in the formulation to introduce proton conduction. The enhancement of proton uptake as a consequence of the introduction of Bi in the formulation was substantiated by DFT simulations and TGA. As further examples of TCO single-phase materials, the double perovskite PBSCF (PrBa_0.5_Sr_0.5_Co_2–*x*_Fe_*x*_O_5+δ_) showed excellent properties as cathode for H-SOFC^118^ and oxygen electrode for H-SOEC.^127^ These properties were attributed, besides the intrinsic triple-conducting characteristics of the compound, to the process of device fabrication. In the former case, a 100 nm thin dense PBSCF layer was deposited by PLD on the BCZYYb electrolytic membrane, and the porous PBSCF cathode was then painted over this PLD base, so ensuring the exchange of reactants due to the porous layer and the closest contact between the cathodic compartment and the electrolyte. In the latter case, the mesh morphology of the PBSCF was obtained by a template derived procedure.^127^ The bundles of hollow PBSCF filaments ensured stability of the oxygen electrode and an optimal accessibility of steam to the electrolyzer. The same mesh morphology was exploited by Ding et al. for PNC (PrNi_0.5_Co_0.5_O_3-δ_). This single-phase TCO material was designed as a modification of the perovskite oxide conductor PCO (PrCoO_3_);^128^ the authors proved by DFT modeling that the insertion of nickel in the B site of PCO produced a drastic decrease of the proton hopping barrier. Mesh-shaped PNC was used to fabricate a reversible proton conducting SOEC-SOFC device that showed stability after prolonged cycling between the SOFC and SOEC modes, high hydrogen yield as SOFC, and high power output as SOEC. The interaction of these single-phase TCO compounds with the respective electrolytes was monitored XRD and/or electron microscopies, also integrated with elemental maps.

In general, a closer inspection of the interfaces involved in the cathodic compartment is performed when triple-conducting oxides are obtained by the formulation of composite materials. These composites can be fabricated by mechanical mixing,^129,19,130,114,6,115,5^ by impregnation of an electrolyte powder by the cathode precursor,^18^ or by one-pot synthesis routes.^112−114,116^ Bi et al.^18^ proposed a composite cathode material obtained by joining the proton conductivity of BZY (BaZr_0.8_Y_0.2_O_3-δ_) with the O^2–^ conductivity and the catalytic activity of SSC (Sm_0.5_Sr_0.5_CoO_3−δ_), a cathodic compound for SOFCs. The SSC nanoparticles were formed in situ by decomposition of the Sm, Co, and Sr nitrates impregnating the BZY particles. As a result, the BZY particles were uniformly decorated by SSC, as demonstrated by the reported SEM-EDS maps. A drawback of this preparation was the growth of the minority phases SrCoO_3_, Co_3_O_4_, and BaCoO_3_, detected by XRD. In particular, the presence of BaCoO_3_ denotes reactivity of SSC with BZY, as observed by Dai et al.,^19^ who prepared SSC-BZY and SSC-BZCY cathodes by mechanical mixing. Shimada et al. assembled anode-electrolyte-cathode cells with composition Ni-BZCYYb | BZCYYb | BZCYYb-LSCF^120^ and Ni-BZCYYb | BZCYYb | BZCYYb-LBC,^130^ using BZCYYb (BaZr_0.1_Ce_0.7_Y_0.1_Yb_0.1_O_3-δ_) as a dense electrolyte and also as a component of both the anode and cathode composites; LSCF and LBC (La_0.6_Ba_0.4_CoO_3−α_) are well-recognized cathodic materials for SOFCs. The FE-EPMA maps relative to the BZCYYb-LBC interface reported no sign of interdiffusion at the device interfaces, while the stability of performance after 60 h operation at 700 °C and 0.5 A cm^–2^ current density was confirmed by EIS.

**Figure 9:**
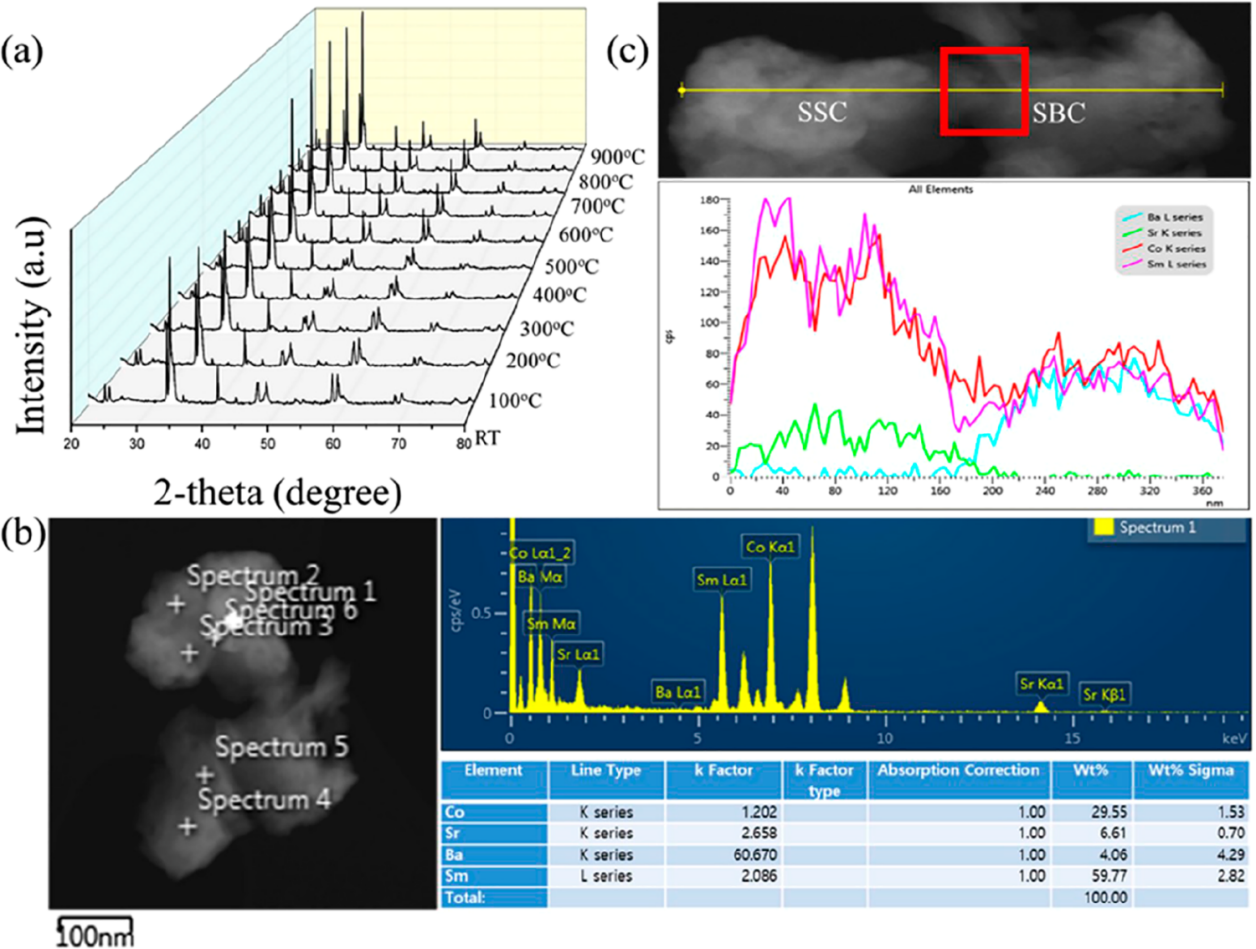
(a) XRD in situ at the indicated temperatures of the SSC-SBC composite. (b) Sample micrograph and STEM-EDX analysis of the composite. (c) Elements distribution along the line drawn across the SSC and SBC grains. Reproduced with permission from ref (5). Copyright 2020 Elsevier B.V.

A different strategy was adopted by Bu et al.^5^ who mixed with the SSC O^2–^ conductor, instead of an electrolytic material, the TCO double layered perovskite SmBaCo_2_O_5+δ_(SBC). The SSC-SBC interaction was monitored by in situ XRD and by STEM-EDX (Figure 9), to check whether the positive effect observed by EIS was due to the growth and synergistic action of an SSC-SBC interface phase. As XRD and STEM-EDX did not evidence the presence of any interfacial phase (in particular the absence (Figure 9c) of interdiffusion between SSC and SBC is worth of notice), a DFT simulation of the interface structure was carried out, leading to the conclusion that an interfacial effect does in fact exist, but of electronic origin, enhancing the oxygen reduction reaction (ORR) activity.

Xie et al. pursued a further different way, mixing two different compounds displaying TCO properties with BCZYYb: the simple perovskite La_0.5_(Ba_0.75_Ca_0.25_)_0.5_Co_0.8_Fe_0.2_O_3-δ_^6^ and the double perovskite Nd(Ba_0.75_Ca_0.25_)Co_1.5_Fe_0.4_Ni_0.1_O_5+δ_ (NBCCFN).^115^ Interestingly, the comparison between two different cathodic composites, BCZYYb-NBCCFN and GDC-NBCCFN,^115^ gave better performance for the latter. The authors concluded that the oxygen reduction reaction (ORR) activity in NBCCFN needed to be improved. This is a reasonable consideration; however, it could also be argued that the interaction between the different components of a SOC, giving the composite peculiar properties with respect to the single components, deserves a specific investigation regarding both the electronic and atomic local structure, as recent research papers^5^ and reviews^131,26^ took into account.

In the composites obtained by one-pot synthesis, usually derived from a Pechini procedure, there is a single precursor that at the end of the preparation route giving rise to two or more different intimately mixed oxide phases. A self-assembled composite cathode was prepared by Song et al.^20^ starting from a nominal composition BaCo_0.7_(Ce_0.8_Y_0.2_)_0.3_O_3−δ_. The final outcome of the synthesis was a composite (P-BCCY)-(M-BCCY), where M-BCCY, with a composition close to BaCo_0.9_(Ce_0.8_Y_0.2_)_0.1_O_3−δ_, is a mixed O^2–^/e^–^ conductor and P-BCCY, with an approximate BaCe_0.8_Y_0.2_O_3-δ_ composition, is a mixed H^+^/e^–^ conductor. The authors report high performance stability after 800 h operation at 750 °C and 0.2–0.3 A cm^–2^ current density of a cell |Ni-BZCYYb|BZCYYb|BCCY| with a peak power density of 700 mW/cm^2^. This very good performance was ascribed to the intimate contact reached by the two cathodic components as a consequence of the self-assembling preparation route, while no evident degradation of performance was evidenced as a consequence of the structural rearrangement of the M-PCCY phase observed by high-temperature XRD and attributed to reduction of Co and to BaCoO_3_ demixing.

**Figure 10:**
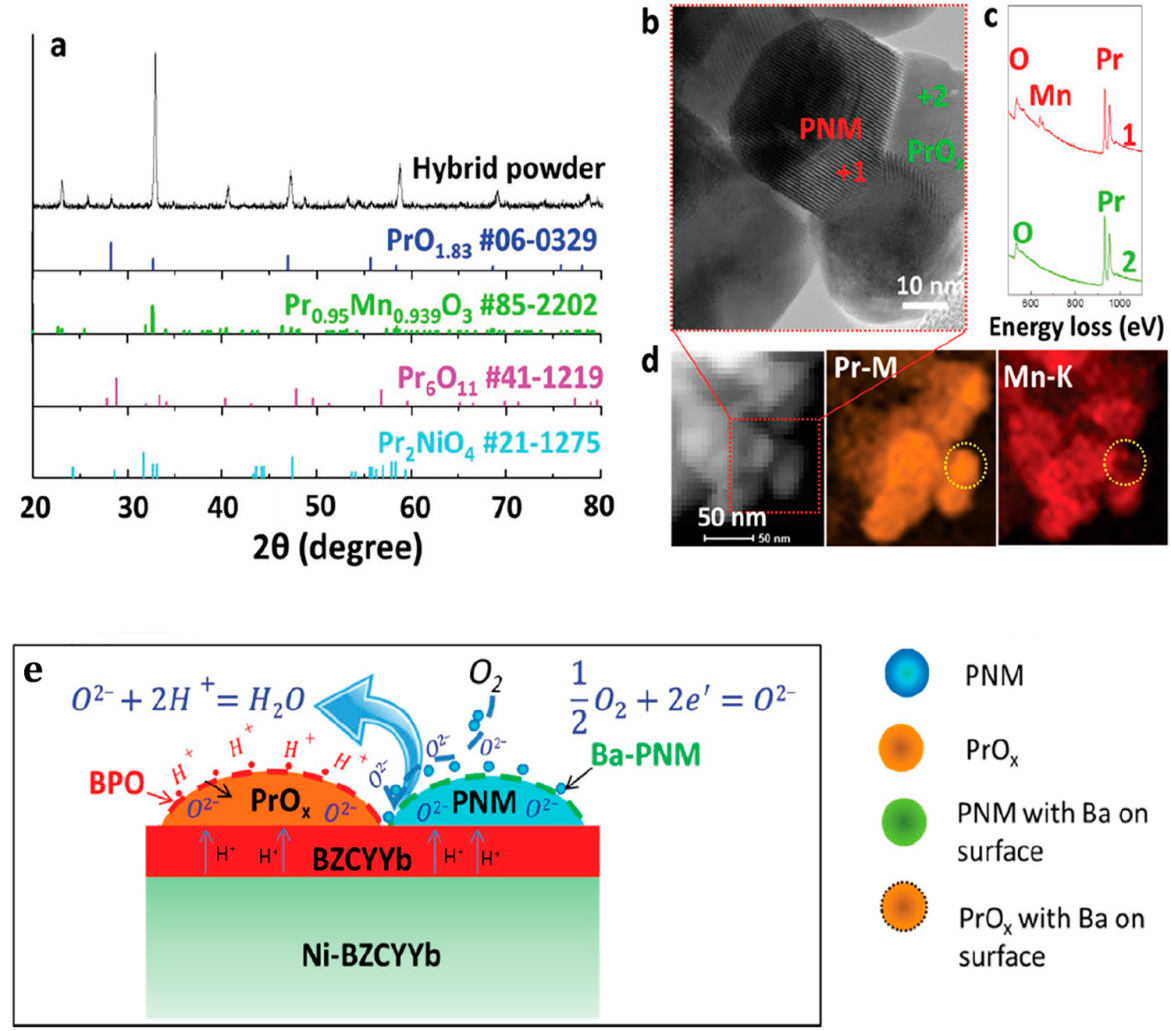
(a) XRD of the composite PNM-PrOx and relevant compounds reference patterns; bright field HRTEM (b) and EELS maps (c) showing the elemental composition of the PNM and PrOx phases; (d) STEM image of the composite powder and EDX maps of Pr and Mn; (e) scheme of the synergic ORR activity between the PNM and the PrOx components. The conjectural formation of a BaPrO_3_ (BPO) proton conducting phase on the PrO_*x*_ surface is outlined. Adapted with permission from ref (112). Copyright 2017 WILEY-VCH Verlag GmbH & Co. KGaA, Weinheim.

Among the composite cathodes prepared with one-pot methods, the case of PrNi_0.5_Mn_0.5_O_3_-PrO_*x*_ (PNM-PrO_*x*_) is particularly interesting.^112^ The material was prepared by a glycine-nitrate process, and its structural and elemental characterization is summarized in Figure 10. The component phases are the fast oxide ion conductor PNM and the highly oxygen-deficient fluorite PrO_*x*_, which is able to increase the ORR rate. The performance of the composite was tested by assembling a Ni-BCZYYb|BCZYYb|PNM-PrOx cell. After a 500 h operation at 700 °C, the XRD lines showed a shift toward lower angles, denoting an increase of the cell size, which was attributed to insertion of a large cationic species, while the XPS spectra revealed the presence of Ba on the cathode surface. The authors argued that a Ba diffusion from the electrolyte toward the cathode took place, producing doping of PNM and growing of a BaPrO_3_ (BPO) surface phase described in the literature as a good proton conductor. The PNM–PRO_*x*_ surface coating by PRO_*x*_ enhances the rate of oxygen reduction reaction and the durability. The proposed mechanism is shown in Figure 10e.

The choice of proton-blocking cathodes, while rarely pursued, has a rationale as it forces the formation of water to be strictly limited at the interface with the electrolyte,^110^ preventing in this way the occupation of reactive ORR sites. On the basis of this consideration, Hou et al. prepared a self-assembled composite cathodic material.^132^ Mixed SDC-PBCu (Pr(Pr_0.5_Ba_1.5_)Cu_3_O_7−δ_) powders were used to prepare an ink painted on the surface of a dense BCZY electrolyte and fired at 900 °C, resulting in a quaternary proton-blocking composite cathode PDC-BCC-SBCC-CuO (Ce_1–*x*_Pr_*x*_O_2−δ_-Ba_2_CeCu_3_O_7.4_-Sm_2_Ba_1.33_Ce_0.67_Cu_3_O_9_-CuO); the high diffusivity of Pr toward SDC, and the counter diffusion of Ce and SM into PBCu, were argued to be at the origin of the solid state reaction yielding the final proton-blocking composite. This was reported to have good stability, tested by 60 h operation at 600 °C. This composite is Co-free, overcoming the problems of Co-containing cathodes (high thermal expansion, easy reduction, evaporation, and high cost), and shows the highest power output (1000 mW cm^–2^ @ 700 °C) among the Co-free cathodic materials. The very complex interface of this composite was observd by HRTEM, but its detailed composition and local structure was not investigated further, except for an XRD bulk characterization.

## 6 In Situ/Operando Studies

In situ/operando investigations in the whole field of solid state electrochemical devices are surprisingly very limited. Besides O-SOC and H-SOC, this remark was raised also for solid state batteries,^133,134^ and the amazement rises from the obvious consideration that correlation with functional performance and a deeper insight into the relevant materials and solid–solid interfaces could be achieved by their observation under operative conditions.

The most exploited techniques in the field of SOCs are XPS, ambient pressure X-ray photoelectron spectroscopy (APXPS), and in situ XAS, as most of the papers are concerned with the cathodic materials under a temperature/reaction environment, sometimes also submitted to an electric bias.^135−137^ So, these studies are focused on the solid–gas interface and are then of marginal interest for this review. Moreover, almost all of the existing literature is relative to oxide-ion conducting devices and their specific materials.

The question of what happens in protonic ceramics in a reactive environment was addressed by Jarry et al.,^138,139^ who investigated by APXPS, complemented with APT measurements, the hydration process of a thin film of BCZY epitaxially grown on MgO. A few other papers reported in situ neutron diffraction experiments carried out at operative temperatures and humid D_2_O atmosphere; the so-obtained structural data relative to Ca-doped lanthanum tungstate, an oxide with proton conduction properties^140^ and In-doped barium zirconate^141^ were correlated to proton conductivity. Nasani et al.^142^ monitored the redox cycling and consequent performance degradation of a Ni-BZY anode composite by environmental scanning electron microscopy (ESEM). These are, to our knowledge, the almost unique examples of application of in situ techniques to the investigation of protonic ceramics.

In this landscape, specific in situ/operando investigations about solid–solid interfaces in protonic ceramics are, to our knowledge, absent. Some hint about the suitability of these studies could come from the ex situ evidence of cation interdiffusion previously cited in this review. Further clues could be drawn, *mutatis mutandis*, from the operando scanning photoelectron microspectroscopy (SPEM) experiments carried out at the Elettra synchrotron on SOFC model devices: for instance, the interaction of the electrode material LSCrM (La_0.75_Sr_0.25_Cr_0.5_Mn_0.5_O_3±δ_) with the oxide-ion electrolyte yttria-stabilized zirconia (YSZ)^143^ was investigated under cathodic polarization, showing the surface diffusion of Sr and Mn from LSCrM onto YSZ and the inverse path from the electrolyte to the electrode under anodic polarization. Similar interdiffusion processes, which could be effective also in protonic ceramic devices at the electrolyte–electrode and at the solid–solid interfaces in composite electrode materials, should deserve suitable in situ/operando investigations in view of further progress in the field. In principle, it could be conceived that under operation conditions, in particular involving the application of an electric bias or the establishment of an electric current loop at high temperature and feeding of reactants, the electronic and atomic structure of the component materials could show peculiar characteristics that should be taken into account for the design of materials and the assemblage of devices. Presently, we have limited evidence in this respect for O-SOCs and only conjectures for H-SOCs.

## 7 Concluding Remarks

The performances of protonic ceramic solid oxide electrochemical devices have shown a definite trend of improvement, that in recent years has begun to substantiate the possibility of their technological exploitation for energy production and conversion. This review is focused on the study of interfaces between the components that constitute the device, since the further improvements in this field are crucially dependent on the detailed knowledge of the atomic structure and properties of interfaces, and on the processes of charge carrier transport and cation interdiffusion taking place at interfaces. In the sections of this paper, we have reviewed in some detail the studies that, to our judgment, constitute good examples of this approach. The first section on electrolytes is mostly dedicated to the analysis of grain boundary structure and composition, from both the viewpoint of experimental analysis at nanometric resolution, and of modellistic ab initio approaches. These investigations are infrequent in the panorama of protonic ceramics, and we believe that a larger amount of information about a wider variety of cases could help in improving materials and devices.

A topic related to the study of grain boundaries is the use of sintering aids, that is explicitly treated in the papers dealing with the improvement of GB conductivity in the most common electrolytes, but also appears in the discussion about the diffusion of nickel from the anode into the electrolytic membrane. These works address various aspects of the fate of sintering aids regarding their oxidation state and/or chemical/structural environment: however, most of the literature tackles this topic with the phenomenological approach of assessing the overall performance of a device. A related question, which to our opinion should be taken into more consideration, is the possible modification of the electrolyte component, due to nickel diffusion, in Ni-electrolyte composite anodes. The topic of anodic composite materials synthesized by exsolution of metal particles from the oxide component is particularly interesting, due to the synergy between these two closely interacting components. Also in this case, following the seminal studies by Irvine and co-workers, detailed investigations of the structure, diffusivity, and oxidation state of the involved cationic species should be suitable.

Studies of interfaces involving cathodes are devoted to the investigation of the interdiffusion in composites, and to the interactions between different phases in some systems grown by in situ techniques. In the case of composite cathodes, the reviewed case studies address the question of the synergistic interaction between the various components and of the interaction of cathodic materials with the electrolyte membrane. The use of single-phase triple conductors is not very frequent, and sometimes such complex oxides are mixed with electrolytes to enhance proton conductivity.

A brief comment should be deserved to the remarkably sporadic investigations of heterostructured electrolytes, which in the field of oxide-ion conducting devices is well represented, with studies of structure and conductivity on the materials and even on the design of specific cell architectures. In-situ/operando studies on protonic ceramic materials are almost absent from the literature, except for the few studies reported in the previous section.

In conclusion, the activity research in the field of protonic ceramics is steadily growing, and interesting performances are being achieved. A deeper insight into the interactions between the different materials constituting the device could promote further improvements. In this review we outlined what are, in our opinion, some good practices and strategies that could be pursued. The first are

(1) local investigation of interfaces, implying an only limited reliance on XRD evidence about stability of interfaces;
(2) computational modellization of interfaces, complementary to experimental approaches;
(3) in situ/operando analysis of materials and interfaces;

and strategies that could be pursued:

(1) exploitation of the strong metal–support interaction, ensuring stability of materials and properties originated by the synergistic interaction of the components, for the electrodes;
(2) engineering of heterointerfaces for electrolytes, involving (once more) computational–experimental investigation of interfaces and study of stability.
